# Abiotic and biotic context dependency of perennial crop yield

**DOI:** 10.1371/journal.pone.0234546

**Published:** 2020-06-26

**Authors:** Thomas P. McKenna, Liz Koziol, James D. Bever, Timothy E. Crews, Benjamin A. Sikes

**Affiliations:** 1 University of Kansas, Lawrence, Kansas, United States of America; 2 The Land Institute, Salina, Kansas, United States of America; Universidade de Coimbra, PORTUGAL

## Abstract

Perennial crops in agricultural systems can increase sustainability and the magnitude of ecosystem services, but yield may depend upon biotic context, including soil mutualists, pathogens and cropping diversity. These biotic factors themselves may interact with abiotic factors such as drought. We tested whether perennial crop yield depended on soil microbes, water availability and crop diversity by testing monocultures and mixtures of three perennial crop species: a novel perennial grain (intermediate wheatgrass—*Thinopyrum intermedium*-- that produces the perennial grain Kernza^®^), a potential perennial oilseed crop (*Silphium intregrifolium*), and alfalfa (*Medicago sativa*). Perennial crop performance depended upon both water regime and the presence of living soil, most likely the arbuscular mycorrhizal (AM) fungi in the whole soil inoculum from a long term perennial monoculture and from an undisturbed native remnant prairie. Specifically, both *Silphium* and alfalfa strongly benefited from AM fungi. The presence of native prairie AM fungi had a greater benefit to *Silphium* in dry pots and alfalfa in wet pots than AM fungi present in the perennial monoculture soil. Kernza did not benefit from AM fungi. Crop mixtures that included Kernza overyielded, but overyielding depended upon inoculation. Specifically, mixtures with Kernza overyielded most strongly in sterile soil as Kernza compensated for poor growth of *Silphium* and alfalfa. This study identifies the importance of soil biota and the context dependence of benefits of native microbes and the overyielding of mixtures in perennial crops.

## Introduction

Perennial crops promise sustainable production and increased environmental benefits relative to annual cropping systems [1]. For example, perennial species allocate more resources to belowground productivity than annuals [2], which may lead to increases in soil carbon, nutrient retention, and hydraulic conductivity [3]. These benefits result from a simultaneous reduction in soil tillage and by shifting the succession of agricultural systems to establish perennial crops that interact with their soil ecosystems for several years or longer [4]. With increased crop longevity and lack of crop rotation, perennials have prolonged interactions with their soil microbiome. For this reason, it is important to understand how perennial crops respond to the biological context of agriculture, including soil pathogens, mutualists, and plant cropping diversity.

Many perennial plants, including perennial crops, are strongly responsive to mutualistic relationships with arbuscular mycorrhizal (AM) fungi [5–7] and are more sensitive to AM fungal identity than annuals [6,8]. Therefore, productivity of perennial crops is likely to be influenced by the composition of AM fungi present in soils. As in annual systems, new perennial plantings typically occur in recently disturbed soils, where land manipulation such as tilling [9–12], crop monocultures [13], and the use of soluble fertilizers and biocides [10,14,15] can lead to degraded AM fungal diversity, composition, and abundance [16]. Past work has shown that new perennial crops benefit from being planted with native AM fungal amendments isolated from undisturbed soils [7].

Productivity of perennial crops may also depend upon interactions with pathogens. While perennials can be better defended against pathogens than annuals [17,18], the longer duration of their plantings makes them more likely to accumulate host-specific pathogens than annual plantings [19,20]. These host-specific pathogens may cause greater declines in productivity over time when compared to annual systems, where rotations of different crops may reduce dominance of crop-specific pathogens [21]. Identifying the relative importance of AM fungal and pathogen components in perennial cropping systems is critical to help leverage plant-microbe interactions for sustained production in perennial agriculture.

Crop diversification, by planting multiple species simultaneously (intercropping), can be an important component for sustainable agriculture that can help mitigate some of the pathogen accumulation as well as abiotic changes predicted to occur with perennial crops [22,23]. Intercropping can increase agricultural productivity, as diversified mixed species plantings commonly have greater yield than monocultures [24,25]. This phenomenon, known as overyielding, is predicted to occur when different crops are able to use different resource pools in space or time [26,27]. Different crops can have disparate growth patterns above and below ground to optimize resource capture (i.e. light, nutrients, and water), which can reduce competition and increase net resource utilization relative to monoculture plantings. For example, the different seasons of activity in wheat and maize intercrops can result in overyielding [28]. This type of overyielding based on reduced resource competition explicitly depends on the availability of specific resources, but the direction of resource effects on overyielding is not always consistent. For example, increasing resource availability can increase [29,30] or decrease overyielding [31]. In addition, crop identity [32], functional group [33], and phylogenetic distance [34] may all be important factors to minimize resource competition, enable facilitation, and create compatible crop mixtures. Thus, understanding species-specific crop companion interactions is essential to predict outcomes and sustainability of crop diversification for landscape scale plantings.

Overyielding via crop diversification can also be mediated by interactions with the soil community [35–37]. Microbial mediation of resource partitioning underlies the classic expectation of overyielding between cereals with high demand for nitrogen and legumes, whose symbiosis with rhizobia allows them to access atmospheric nitrogen [37,38]. In addition, symbioses with AM fungi can ameliorate resource deficiencies for hosts (nutrients [39–41] and water [42–44]) and can alter the strength of interactions between species [45]. For example, AM fungi mediation of resource partitioning is supported by increased complementarity observed between maize and faba bean when in association with AM fungi [37]. Soil pathogens could also mediate overyielding, as accumulation of species-specific pathogens may limit yield in monoculture plantings [46]. Substitutive planting with another crop lowers host density in mixture, resulting in decreased pathogen abundance and a reduction in this deleterious effect [35,46–48].

While both pathogen accumulation and microbially-mediated resource partitioning have been observed to generate overyielding in perennial [37,47,49] and annual [37] systems, these overyielding mechanisms themselves may be context dependent [50]. The benefits of intercropping legumes may be reduced in soils with high nitrogen availability [31], and the positive effect of AM fungi on overyielding may be decreased when phosphorus is abundant [37]. While AM fungi mediated impacts on overyielding under different levels of water availability are less known, pathogen impacts on hosts do vary with water availability [51]. This could cause the magnitudes of overyielding to vary between wet and dry conditions. Understanding the biotic and abiotic contexts and mechanisms driving overyielding can help predict compatible perennial crop pairs, and potentially illuminate ways to increase sustainability of perennial crop plantings.

The objective of this experiment was to determine the compatibility and potential overyielding in mixtures of three perennial crop species under different abiotic (water availability) and biotic (changes in soil biota) conditions. We chose perennial crop candidates that have cereal, oilseed, and forage production potential and also represent three distinct functional groups (cool-season grass, forb, and legume). The cool-season grass *Thinopyrum intermedium* produces the novel perennial grain Kernza^®^ and it has been selected for many desired agronomic traits at The Land Institute in Salina, KS [52]. Throughout the rest of the manuscript, Kernza will be used to describe the entire crop plant not just the grain. The forb *Silphium integrifolium* (Rosinweed) is also being studied at The Land Institute. It is a warm -season forb native to the tallgrass prairie and has potential as a perennial oil seed crop [53]. The commonly farmed alfalfa (*Medicago sativa*) was used as the perennial legume. Mixtures of these species have the potential to increase a number of ecosystem services [4,22], yet more research is needed to understand the interactions of these crops under different abiotic and biotic contexts. In this greenhouse study, we ask these questions to better understand the interactions of these crops:

1. How do the planted crop community, soil community, and water availability influence the performance of the perennial crop species?
2. Do mixtures of these perennial crops overyield relative to their component monocultures?
3. Is any overyielding mediated by the soil community, water availability, or their interaction?

## Materials and methods

### Experiment location

The pot experiment was conducted in the west campus greenhouse at the University of Kansas in Lawrence, Kansas U.S.A. Greenhouse temperature controls were set to allow a temperature range of 65 to 85°F and no supplemental lighting was used.

### Soil inoculum

Pots (7 L) were partially filled with a steam sterilized (twice at 174° F) 50:50 sand:soil mixture. The nutrient content of the sterilized soil was 15.8 ppm phosphorus via Melich extraction and 26.55 ppm nitrate (NO3-N) and 5.8 ppm ammonium (NH4-N) via KCl extractions. One of four soil inoculum was added (280 cm^3^ total, 4% by volume), and then the pots were filled the rest of the way with the sterile sand:soil mixture. Each inoculum consisted of two components (140 cm^3^ each): live whole soil and live prairie AM fungi (LWLF), live whole soil and sterilized prairie AM fungi (LWSF), sterile whole soil and live prairie AM fungi (SWLF), or sterile whole soil and sterile prairie AM fungi (SWSF). The small volume of inoculum was used to minimize potential differences in abiotic properties among the inoculum, which may be due to soil conditioning effects or nutrient release after sterilization [54].

The whole soil (LW) was collected from long-term (established in 2002) monoculture plots of intermediate wheatgrass (*T*. *intermedium*) at The Land Institute in Salina, KS as part of the Agroecology Research Trials (38.767690°, -97.572539°). We chose to use a soil community with a history of long-term soil conditioning by *T*. *intermedium*, without disturbance (no tillage), to test *T*. *intermedium-*specific pathogens and mutualists (i.e. AM fungi), which have been shown to be important in mediating overyielding in perennial systems [37]. Whole soil was collected from the top 10 cm, sieved (1 cm), and stored at 4°C for less than one week prior to inoculating the experiment.

The prairie AM fungi inoculum was isolated and cultured from a native Kansas remnant prairie (39.044991°, -95.191569°) with Oska silty clay loam and Pawnee clay loam soil types [55]. Undisturbed remnant prairies contain unique AM fungi communities not found in highly disturbed agricultural systems [16], and studies have shown differential responses of plant species to fungi isolated from remnant prairies relative to disturbed fungi [6]. In a previous experiment, alfalfa and *Silphium* were shown to be highly responsive to AM fungi [7]. This inoculum was used to test the differential responsiveness of the crop communities to the whole soil and prairie AM fungi inoculum. The prairie AM fungi inoculum consisted of seven AM fungi species with high spore abundance at the time of sampling: *Scutellospora dipurpurescens*, *Gigaspora gigantea*, *Funneliformis mosseae*, *Funneliformis geosporum*, *Glomus mortonii*, *Rhizophagus diaphanous*, and *Claroideoglomus claroideum*. Each species of AM fungi was cultured independently on native prairie plants for one growing season in a sterilized 50:50 sand:soil mixture (10.15 ppm P via Melich extraction, 7.375 ppm NO3-N, 22.2 ppm NH4-N via KCl extractions) under greenhouse conditions (see [56] for a detailed description of isolation and culturing). A community mixture of these cultures was homogenized and used as our native AM fungi treatment ("LF" for living cultures). All biota from the live whole soil and live fungi were sterilized via autoclaving (2 X 60 minutes at 121°C) to create the sterile whole soil (SW) and sterile AM fungi (SF) treatments so that each pot had similar additions of whole soil and cultured fungal inoculum, whether living or dead. The sterilized SWSF inoculum was used to test the responsiveness of the crop communities in the absence of soil biota.

### Crop community

Six crop communities were designed to test overyielding that included all possible combinations of monoculture and biculture plantings for the three perennial crop species, *Silphium integrifolium* (henceforth referred to as *Silphium* or S), *Medicago sativa* (henceforth referred to as alfalfa or A) and *Thinopyrum intermedium* (henceforth referred to as Kernza or K). Any combination of two letters represents a biculture (i.e. KA represents a Kernza/alfalfa biculture plant community). Kernza and *Silphium* seeds were obtained from The Land Institute’s breeding program, and The Land Institute granted permission for seed use. Alfalfa (Kansas Common variety) seeds were purchased from a commercial supplier. *Silphium* seeds were cold moist stratified two months prior to germination. Alfalfa was inoculated with commercially produced rhizobia (Exceed Superior Legume Inoculant for alfalfa/true clover, Visjon Biologics, Wichita Falls, TX, USA). Seeds of all crop species were germinated and grown for one week at the end of March in 2018 on a sterilized (2 X autoclaved as above) sand:soil mixture. We planted four conspecific seedlings (one week old) into each pot for monocultures, and two conspecific plants were planted diagonally from each other in each biculture.

### Water availability

Pots were randomized via split block where half the block was well-watered, and the other half was given a drought treatment. All plants were well-watered for 18 days before drought treatments were applied by watering twice daily for two minutes (266.7 ml/day) via a drip irrigation emitter to prevent splashing of soil microbes. On day 19, drought pots were watered twice per day every other day for 1 minute (133.3 ml/day), while well-watered pots received no change in water volume for the duration of the experiment. The full experiment design included 7 replicates of each crop community, water regime, and inoculation combination (2 levels of water treatment x 6 levels of crop community x 4 levels of inoculum x 7 replicates = 336 pots).

### Data collection

Crops were grown for 8 weeks, and then aboveground biomass was collected by cutting at 4 cm above the soil surface line, separated to species, dried at 60° C, and weighed. Crops were allowed to regrow an additional 5 weeks and a second harvest was performed. A second harvest was conducted to assess the context dependency of biotic and abiotic effects on crop regrowth, as aboveground biomass of perennial systems may be cut multiple times in one growing season [4,22]. Ten plants out of 1344 (0.7%) died before the second harvest. These plants were recorded as 0.0 g at harvest 2.

After the second harvest, root tissues were collected from a subset (4 blocks) of the monoculture pots to confirm AM fungal colonization. Root subsamples from each pot were cleared and stained with Trypan Blue. Hyphae and arbuscules were counted using the magnified intersections method [57]. The results from the root analysis showed that the presence of AM fungal hyphae and arbuscules was greater in monoculture pots with live soil inoculum (LWLF, LWSF, and SWLF) than in pots inoculated with sterile whole soil and sterile prairie AM fungi (SWSF) (See supporting information for detailed results; S1 Table; S1–S3 Figs). The mean hyphal and arbuscule presence for the sterile inoculum (SWSF) was close to zero.

### Statistical analyses

#### Crop-specific responses

To gain insight on the crop-specific responses and uncouple a three way interaction between the water, inoculum, and crop species nested within crop community treatments (see S2 Table), a separate mixed model for each crop was analyzed with yield per individual as the response and block, water, crop, and inoculum set as fixed factors in SAS (proc mixed, SAS v9.4, SAS Institute, Cary, NC, USA). To account for the spatial separation of the watering treatment within each block, a block x water interaction and its’ higher order interactions were included as random effects. Yield per individual was natural log transformed to meet statistical assumptions. Tukey’s HSD multiple comparisons test was used to determine differences among groups within a significant treatment effect. Because results were similar for each harvest response (S2 Table), only total harvest responses are presented.

#### The effects of water, crop community, and inoculum on overyielding

For analysis of overyielding, we calculated the average individual yield of each crop species in each pot. We used this as a response in a mixed model (proc mixed) in SAS (version v.9.4, SAS institute, Cary, NC, USA) with pot designated as the subject. Average individual yield was natural log transformed to meet statistical assumptions. Block, water, crop community, crop species nested within crop community, and inoculum treatments were designated as fixed factors. The block x water x crop x inoculum x pot interaction was included as a random effect to account for multiple samples taken from the same pot (in mixtures), and the block x water interaction and its’ higher order interactions were included as random terms to account for the spatial separation of the watering treatment within each block. To test for overyielding and its abiotic and biotic context dependency, we designed four set of contrasts to compare monoculture vs mixture performance within each crop community, inoculum, and water combination. There were four sets of contrasts: 1) all possible combinations of mixtures versus component monocultures overall and for all three possible combinations of crop community designs (KA, KS, AS), 2) all possible combinations of the overall and crop species specific interaction of mixtures versus monocultures when grown among living soil (LWLF, LWSF, SWLF vs. SWSF), AM fungi (LWLF, SWLF vs LWSF, SWSF), or whole soil (LWLF, LWSF vs. SWLF, SWSF), 3) all possible combinations of the overall and crop species specific interaction of mixtures versus monocultures by water treatment, and 4) all possible combinations of the overall and crop species specific interaction of mixtures versus monocultures when grown among living soil, AM fungi, or whole soil by water treatment. We analyzed crop performance with data from the first and second harvest as well as the combined harvests for a total harvest. Results were similar for the first, second, and total harvest (S3 Table), so here we present total harvest results only.

## Results

### Crop-specific responses to crop community, inoculum, and water

**Fig 1:**
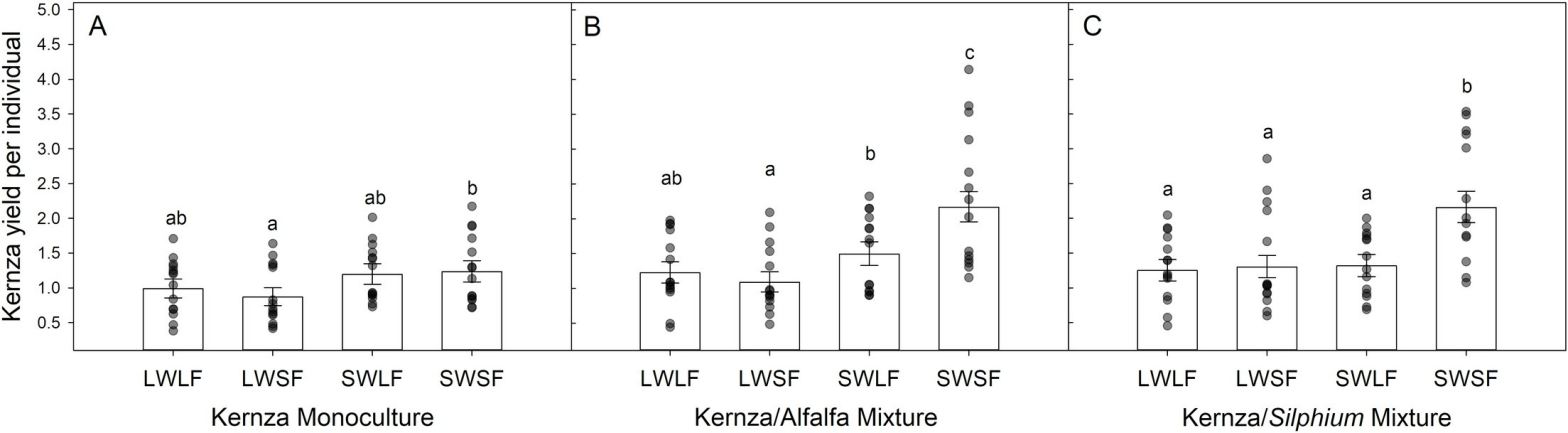
Average yield per individual Kernza biomass (back transformed LS Mean grams ± 95% Confidence Limits) in pots with the four soil inoculation treatments (LW=living whole soil, SW= sterilized whole soil, LF= living prairie AM fungi, SF=sterilized prairie AM fungi) when Kernza was planted in monoculture (A), with alfalfa (B), and with *Silphium* (C). The dots are the observed responses for each treatment. Bars with different letters within each cropping treatment are significantly different (Tukey’s HSD multiple comparison).

Kernza growth was 25% better in mixture than monoculture (crop treatment main effect; Table 1), and the growth of Kernza was inhibited 26 – 30% by the presence of live whole soil and live prairie AM fungi relative to sterile soil (inoculum treatment main effects; Table 1). Increasing water availability increased Kernza growth by 50% (water treatment main effect; Table 1). Kernza performed the best when planted in mixture with sterile soil (SWSF) inoculum (crop x inoculum interaction; Table 1; Fig 1), and removal of prairie AM fungi (LWSF) only significantly decreased growth relative to removal of whole soil (SWLF) when Kernza was planted with alfalfa in wet pots (water x inoculum x crop interaction; Table 1)

**Table 1:** Degrees of freedom (numerator (Num) and denominator (Den)), F value, and p value from analysis of the effect of water, crop community, and soil inoculum on total harvest yield per individual of each crop (Kernza, alfalfa, and *Silphium*).

		Kernza			*Silphium*			Alfalfa		
Effect	Num	Den	F	P	Den	F	P	Den	F	p
Block	6	42.1	4.16	0.0023	6.00	1.43	0.3384	6.01	0.56	0.7479
Water (W)	1	42.1	162.27	<0.0001	6.02	102.80	<0.0001	6.01	82.27	<0.0001
Crop community (C)	2	95.3	55.54	<0.0001	97.9	4.92	0.0092	24.2	2.23	0.1292
Inoculum (I)	3	42.1	24.71	<0.0001	36.5	224.49	<0.0001	35.2	211.93	<0.0001
W x C	2	95.3	0.18	0.8381	98.0	2.08	0.1300	24.2	3.91	0.0336
W x I	3	42.1	1.03	0.3896	36.5	27.87	<0.0001	35.2	52.45	<0.0001
I x C	6	95.3	7.33	<0.0001	97.7	0.57	0.7519	70.1	1.89	0.0942
W x I x C	6	95.3	2.82	0.0143	97.7	0.29	0.9385	70.1	1.91	0.0920

**Fig 2:**
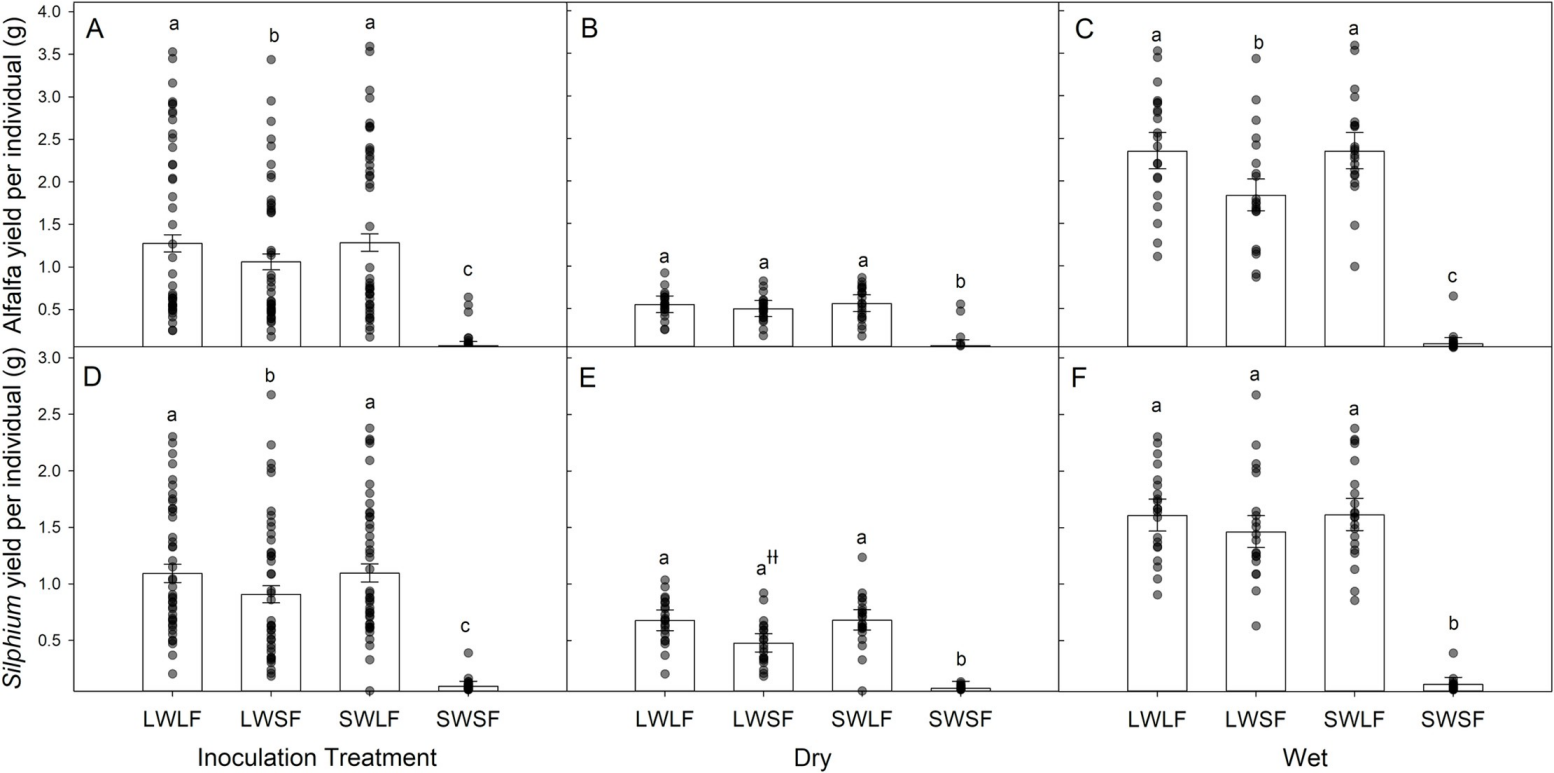
Average yield per individual biomass (back transformed LS Mean grams ± 95% Confidence Limits) of alfalfa (A - C) and *Silphium* (D – F) in the soil inoculation treatments (A and D; LW=living whole soil, SW= sterilized whole soil, LF= living prairie AM fungi, SF=sterilized prairie AM fungi) and in the soil inoculation treatments with dry (B and E) and wet (C and F) water treatments. The dots are the observed responses for each treatment. Bars with different letters within each graph are significantly different (Tukey’s HSD multiple comparison). ^ƚƚ^ The Tukey’s comparisons of LWSF vs LWLF and LWSF vs SWLF in panel E were marginally significant (P = 0.06) but the interaction of water x inoculum (Panel E vs F) was substantially significant (P <0.0001; Table 1).

*Silphium* growth was reduced 11% in mixture with Kernza relative to being planted in monoculture (crop treatment main effect; Table 1), and alfalfa growth was reduced by 34% in mixture with Kernza in dry pots (water x crop interaction; Table 1). Alfalfa and *Silphium* had greater growth (increases ranging from 600 to 1000%) in the presence of whole soil (LWSF) and live prairie AM fungi (SWLF) or their combination (LWLF) relative to non-inoculated (SWSF) (inoculum main effects; Table 1; Fig 2A and 2D). Increasing water availability increased alfalfa growth by 163% and *Silphium* growth by 95%. Water availability also moderated inoculum effects on alfalfa and *Silphium* growth (water x inoculum interaction; Table 1). Inoculation with native prairie AM fungi (LWLF and SWLF) increased alfalfa growth most in wet pots (Fig 2B vs 2C), and increased *Silphium* growth most in dry pots (Fig 2E vs 2F).

### Overyielding depends on crop pairs

**Fig 3:**
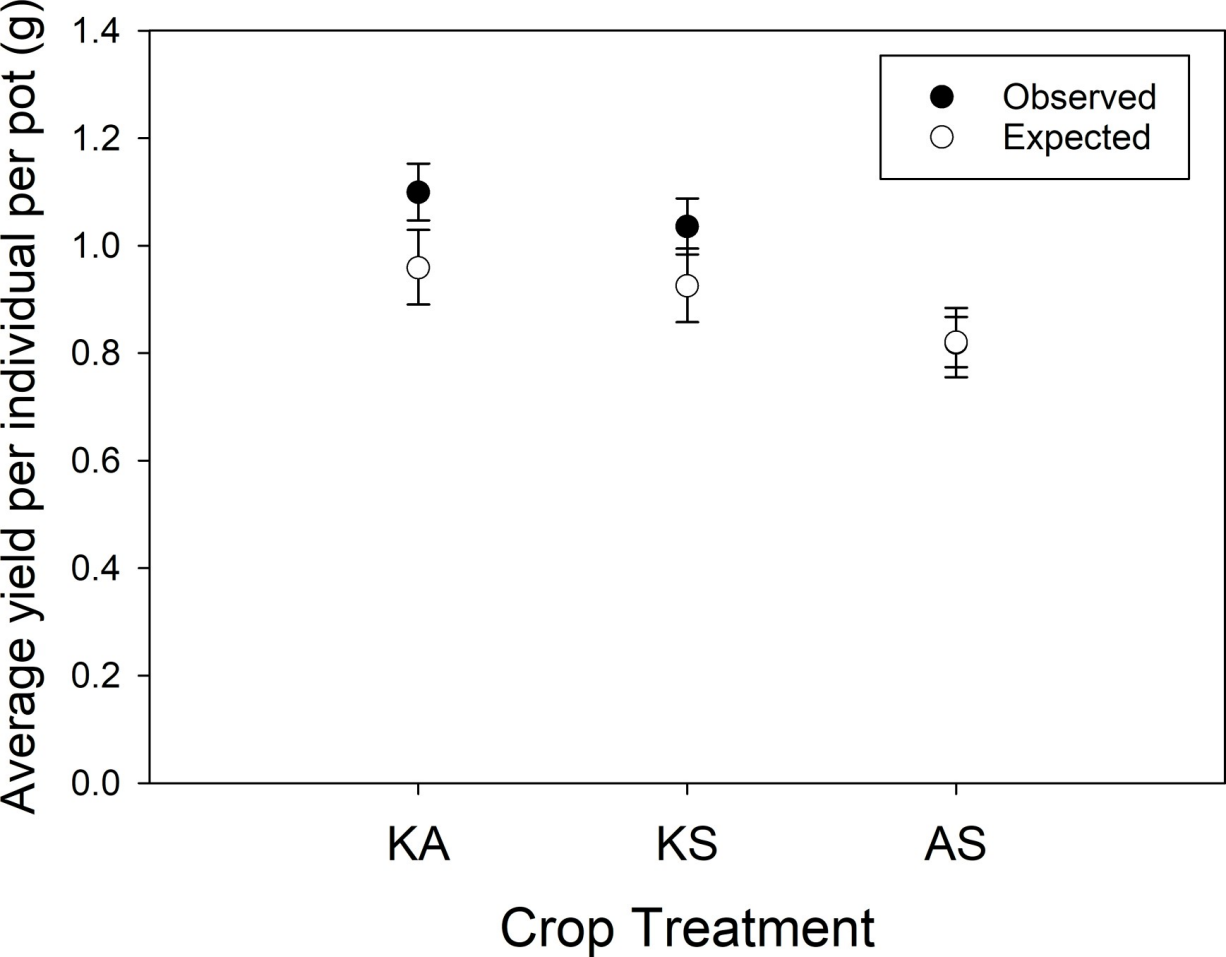
Average observed and expected (average yield of the component monocultures) yield per individual (back transformed LS Mean grams ± 95% Confidence Limits) for each crop mixture (A=alfalfa, K=Kernza, and S=*Silphium*).

Crop mixtures overyielded relative to monocultures (P = 0.0004; contrast set 1; Overall; Table 2), but the level of overyielding depended significantly on the crop pairing (P < 0.0001; contrast set 1; Mix vs mono x crop community; Table 2). Comparing each crop community (KA, KS, AS) individually to their respective monoculture components, we found significant crop community overyielding for KA (Fig 3, 10.5% overyielding, P <0.0001; contrast set 1; KA) and KS (Fig 3, 8.9% overyielding, P = 0.0006; contrast set 1; KS). Crop communities of AS did not overyield (P = 0.9829; contrast set 1; AS).

**Table 2:** Degrees of freedom (numerator (Num) and denominator (Den)), F value, and P value, for planned contrasts to test for overyielding in mixtures (mix) relative to component monocultures (mono) of Kernza (K), alfalfa (A), and *Silphium* (S) with different soil inoculum present. Live represents the presence of native AM fungi, whole soil, or both.

Contrast Set	Contrast	Num DF	Den DF	F Value	P
1	mix vs mono overall	1	384	12.85	0.0004
1	mix vs mono x crop community	3	385	8.71	<0.0001
1	mix vs mono KA	1	382	17.45	<0.0001
1	mix vs mono KS	1	384	11.85	0.0006
1	mix vs mono AS	1	388	0.00	0.9829
2	mix vs mono overall x whole soil	1	384	0.96	0.3272
2	mix vs mono overall x AM fungi	1	384	2.51	0.1142
2	mix vs mono x live vs sterile	1	383	6.46	0.0114
2	mix vs mono KA x whole soil	1	382	0.07	0.7850
2	mix vs mono KA x AM fungi	1	382	1.56	0.2126
2	mix vs mono KA*live vs sterile	1	382	4.25	0.0398
2	mix vs mono KS x whole soil	1	384	2.16	0.1429
2	mix vs mono KS x AM fungi	1	384	7.43	0.0067
2	mix vs mono KS x live vs sterile	1	387	12.73	0.0004
2	mix vs mono AS x whole soil	1	388	0.11	0.7407
2	mix vs mono AS x AM fungi	1	388	0.39	0.5343
2	mix vs mono AS x live vs sterile	1	386	0.10	0.7513

**Fig 4:**
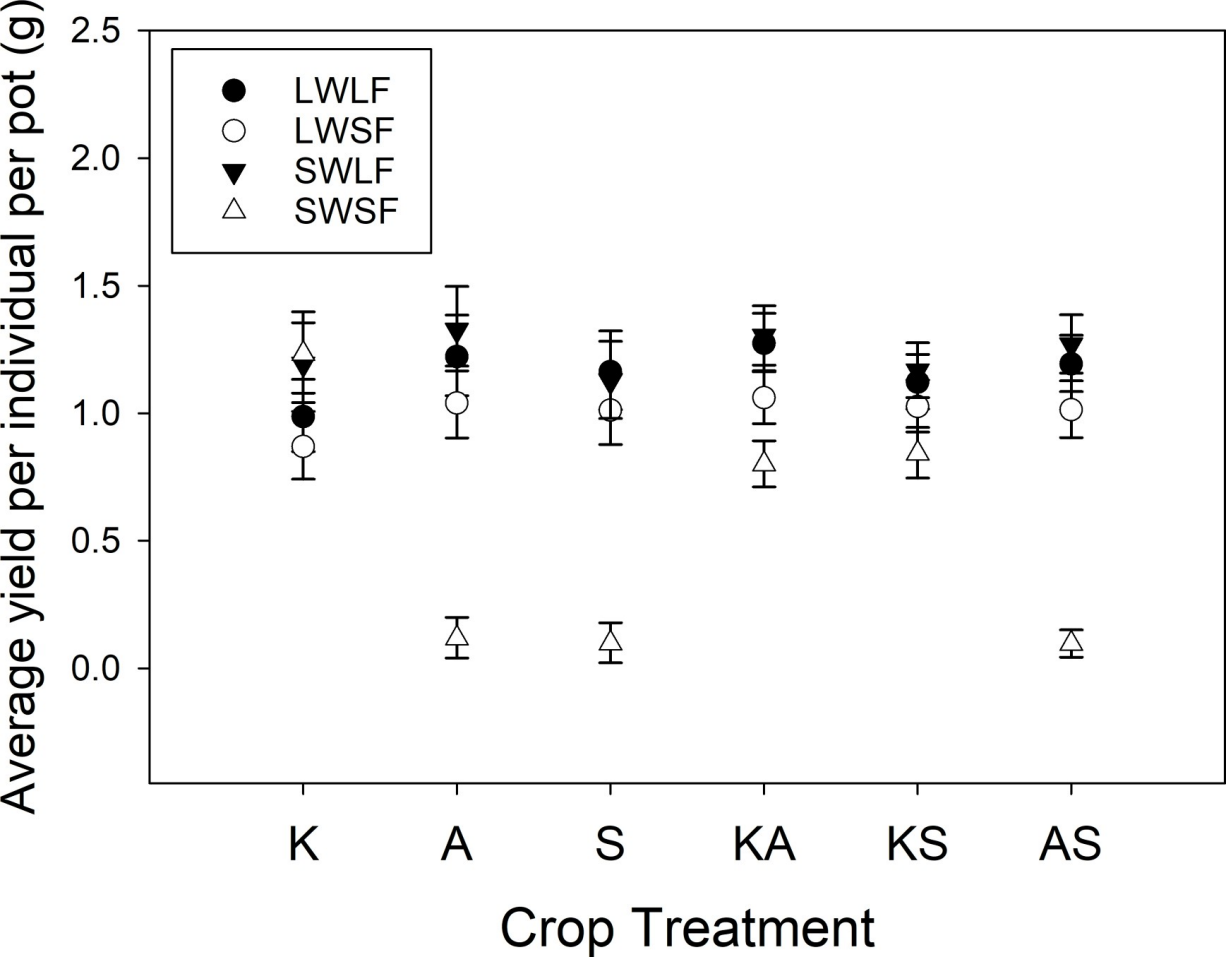
Average yield per individual per pot biomass (back transformed LS Mean grams ± 95% Confidence Limits) in each crop community (K=Kernza, A=alfalfa, and S=*Silphium*) and inoculation treatment (LW=living whole soil, SW= sterilized whole soil, LF= living prairie AM fungi, SF=sterilized prairie AM fungi).

The presence of living inoculum (LWLF, LWSF, SWLF) vs sterile inoculum (SWSF) had significant effects on overyielding across crop communities (contrast set 2; Mix vs mono x live vs sterile (P = 0.0114)) and in crop specific mixtures (contrast set 2; Mix vs mono x live vs sterile KA (P = 0.0398), KS (P = 0.0004), and AS (P = 0.7513)). Living inoculum substantially reduced overyielding in mixtures of KA (61% reduction) and KS (86% reduction), but not in mixtures of AS. When looking at the effects of specific inoculum, AM fungi (LWLF, SWLF vs LWSF, SWSF) significantly lowered overyielding in mixtures of KS (87% reduction; P = 0.0067; contrast set 2; Mix vs mono KS x AM fungi), while no significant effects were found for whole soil inoculated pots (LWLF, LWSF vs SWLF, SWSF; contrast set 2; Mix vs mono x whole soil Overall, KA, KS, AS). We found no significant contrasts for any combinations of AS by inoculation treatment, but in general, A and S monocultures performed extremely poorly without living biota (SWSF; Fig 4). Crop community and inoculum effects on overyielding were consistent across watering treatments (S2 Table; contrast set 3 and 4), except for a marginal 11.6% increase in KA overyielding in wet pots relative to dry pots (P = 0.0583; contrast set 4; KA).

## Discussion

We found very strong effects of inoculation, watering regime, and plant diversity on crop productivity, and that these effects varied markedly across the crop species. Notably, we found that two crops, *Silphium* and alfalfa, were very responsive to the presence of arbuscular mycorrhizal (AM) fungi. Each of these species benefited more from native AM fungi, but this benefit depended on water availability. While native AM fungi was particularly beneficial to *Silphium* in drought conditions, native AM fungi benefited alfalfa most under well-watered conditions. In contrast, Kernza did not benefit from AM fungi and grew best in sterile soil. Crop mixtures that included Kernza overyielded, but this overyielding depended on the presence of soil biota (live vs sterile). Unlike previous work in annual [35,37] and perennial [36,47,49] systems, overyielding in our system was greatest in sterile soil, where Kernza compensated for the poor growth of *Silphium* or alfalfa. While water availability had large effects on total productivity, it had only marginal effects on overyielding. These results reinforce the importance of soil biota, especially AM fungi, in crop-specific performance and overyielding.

### Crop-specific responses to water and inoculum

Both biotic and abiotic context strongly affected the growth of alfalfa and *Silphium*, especially the presence of AM fungi. While the whole soil inoculum increased growth, both species tended to perform better in the presence of native prairie AM fungi. Other studies have shown that AM fungi dependent plant species perform better with inoculations of AM fungi from locally adapted undisturbed systems [7,58]. This is because AM fungal communities in agricultural systems tend to differ in composition and be less beneficial following anthropogenic manipulations such as crop tillage and chemical application [12,15]. It should be noted that our whole soil inoculum contained all components of the soil community, including AM fungi, bacteria, nematodes, pathogens, etc. Thus, the reduced benefit found for alfalfa and *Silphium* for whole soil versus prairie AM fungi could be attributed to less beneficial AM fungi and/or the presence of these other soil biota inhibiting crop productivity. Regardless, this work suggests that native, locally adapted mycorrhizal amendments may boost the growth of mycorrhizal-dependent plant species in perennial agricultural plantings. Future work should isolate the effects AM fungal composition and the broader microbiome in promoting perennial cropping systems.

Not only was the growth of our mycorrhizally sensitive crop species dependent on soil inoculum composition, but the response of alfalfa and *Silphium* to the presence of whole soil biota varied with water availability. We expected the presence of AM fungi to boost plant resistance to drought conditions [42–44]. *Silphium* supported this pattern as it performed better with native AM fungi in water limited conditions. In contrast, native AM fungi increased alfalfa performance in wet conditions. This could be linked to native AM fungi enhancing facilitation of phosphorus uptake, and phosphorus being more limiting in well-watered conditions. However, the contrasting effects of water on AM fungal inoculation effects further highlights the importance of abiotic and biotic context dependency in polyculture systems. Apart from AM fungi effects depending on water, effects of pathogens present in whole soils may also vary with water since pathogens often proliferate under well-watered conditions [59]. Based on this we expected susceptible plants to perform more poorly with whole soil biota under well-watered conditions. However, we did not observe growth inhibition due to pathogen accumulation under well-watered conditions in this study.

Despite our whole soil inoculum being sourced from a long-term field trial of the Kernza progenitor, the lack of responsiveness to soil biota—positive or negative—may be attributed to Kernza being a mid-successional introduced cool-season grass. Mycorrhizal responsiveness tends to increase with plant successional stage [17,60], is stronger for native than non-native plant species [8], and C_3_ grasses (cool-season) are less responsive than other plant functional groups [5]. Thus, we anticipated that Kernza would not demonstrate strong mycorrhizal responses. Past work has shown a lack of or reduction in mycorrhizal responsiveness for introduced plant species [8,61], and this difference may grant novel crops an edge as they are introduced into new agricultural environments. Novel environments may also give introduced crop species an edge because they may also be less susceptible to species-specific pathogens because the pathogens may not have been co-introduced with the host [62].

### Crop-specific responses to abiotic and biotic conditions resulting in overyielding

While previous studies have found evidence of pathogen-mediated overyielding in annual [37] and perennial systems [36,47,49], we did not find support for this mechanism in this system. In retrospect, this might not be surprising, as our soil collection targeted potential pathogens of Kernza by using inocula from a mature Kernza field, but Kernza is a relatively newly introduced species in Kansas and newly introduced plant species often do not suffer negative effects of host-specific pathogens [62,63]. Moreover, the soil from the Kernza field may not have abundant host-specific pathogens of *Silphium* or alfalfa. Both *Silphium* [64] and alfalfa [65] do suffer heavy losses from host-specific pests in the mid-western US, and it is possible that pathogen mediated overyielding could have been observed with a different initial soil inoculum. Moreover, overtime non-native plant species accumulate pathogens [66] and therefore, as Kernza is planted more widely, intercropping may become important to managing pathogen accumulations and sustaining Kernza yield in the future.

While individual studies have found evidence for mycorrhizally mediated overyielding [36,37], several studies have found less overyielding with AM fungi alone compared to whole soil [36,47]. In our case, we did not find overyielding with AM fungi, but did find overyielding in sterile soil in mixtures that include Kernza, as Kernza compensated for the very poor growth of the AM-dependent *Silphium* and alfalfa. This compensation was largely independent of water treatment. This context dependence is not consistent with prior expectations of AM mediation of overyielding. Moreover, we did not see evidence of symbiotic N-fixation mediating overyielding in mixtures that include legumes. This is surprising given that it is a commonly invoked mechanism of microbially-mediated resource partitioning and facilitation [32,67,68]. Longer experiments including those in the field may have generated greater N-limitation and more context for symbiotic N facilitation [4], as enhanced benefits of polycultures of Kernza and alfalfa may take as long as four years to develop [69].

This study reinforces that soil biotic effects on perennial polycultures are context dependent and gives insight into interactions among specific perennial crops. Longer term field studies and studies that include potential host-specific beneficial and pathogenic microbes of all crops would enhance our understanding of overyielding in perennial systems, particularly because the relative importance of biotic and abiotic factors may change over time. Given the life cycle of perennial crops and our ultimate goals for sustainable production, long term monitoring is even more essential than in annual systems.

### Perennial polycultures as a model for future cropping systems

Our study found consistent yield across the 6 different crop communities treatments, whether crops were grown in mixture or monoculture. While overyielding was only found in sterile soil conditions that are absent in the field, our work suggests that bi-culture plantings can result in similar field production yields as monocultures, while providing other beneficial ecosystem services. For instance, incorporating a companion crop such as alfalfa or *Silphium* can improve pollinator abundance, increase forage and habitat quality, or create a new revenue stream [22]. Moreover, these consistent yields across planting were also present at each level of water availability. So although well-watered plants grew better than drought plants, we found that bi-cultures persisted and provided as much mass as monocultures when water was limited. The findings of our study should also be considered in new plantings when agricultural landscapes are converted from annual systems to perennial systems. Although we did not find strong evidence of overyielding due to biotic conditions, polycultures with the highest per capita yields tended to be inoculated with whole soil and native mycorrhizal amendments (Fig 2). These data highlight that choosing or manipulating the biotic conditions to meet the needs of plant species grown together can help achieve the greatest yields when planting of future polyculture perennial crops.

## Supporting information

S1 Table Degrees of freedom (numerator (NUM) and denominator (Den)), F value, and p value from analysis of the effect of water, crop species (identity), and soil inoculum on the presence of AM fungal hyphae and arbuscules. (DOCX)

S2 Table Degrees of freedom (numerator (NUM) and denominator (Den)), F value, and p value from analysis of the effect of water, crop community, crop species nested within crop community, and soil inoculum on total average yield per of each crop (Kernza, alfalfa, and *Silphium*) in the first, second, and total harvest. (DOCX)

S3 Table **Degrees of freedom (numerator (Num), denominator (Den)), F value, and P value, for planned contrasts to test for overyielding in mixtures (mix) relative to component monocultures (mono) of Kernza (K), alfalfa (A), and *Silphium* (S) with different soil inoculum present and changing water availability for the first, second, and total harvest.** Live represents the presence of native AM fungi, whole soil, or both.
(DOCX)

S1 Fig Hyphal colonization (back transformed LS mean ± 95% confidence limits) in monoculture pots with differing watering treatments (A), crop identities (B), and soil inoculation treatments (C). Bars with different letters within each graph are significantly different (Tukey’s HSD multiple comparison).
(TIF)

S2 Fig Hyphal colonization (back transformed LS mean ± 95% confidence limits) in monoculture pots with differing water treatments and crop identities. Bars with different letters within each graph are significantly different (Tukey’s HSD multiple comparison).
(TIF)

S3 Fig Arbuscule presence (back transformed LS mean ± 95% confidence limits) in monoculture pots with differing crop identities (A) and soil inoculation treatments (B). Bars with different letters within each graph are significantly different (Tukey’s HSD multiple comparison).
(TIF)

S1 File (DOCX)

## References

[1] Pimentel D, Cerasale D, Stanley RC, Perlman R, Newman EM, Brent LC, et al Annual vs. perennial grain production. Agric Ecosyst Environ. 2012; 161: 1–9.

[2] Vico G, Manzoni S, Nkurunziza L, Murphy K, Weih M. Trade-offs between seed output and life span – a quantitative comparison of traits between annual and perennial congeneric species. New Phytol. 2016; 209: 104–114. 10.1111/nph.13574 26214792

[3] Crews TE, Carton W, Olsson L. Is the future of agriculture perennial? Imperatives and opportunities to reinvent agriculture by shifting from annual monocultures to perennial polycultures. Glob Sustain. 2018; 1.

[4] Crews TE, Blesh J, Culman SW, Hayes RC, Jensen ES, Mack MC, et al Going where no grains have gone before: From early to mid-succession. Agric Ecosyst Environ. 2016; 223: 223–238.

[5] Hoeksema JD, Chaudhary VB, Gehring CA, Johnson NC, Karst J, Koide RT, et al A meta-analysis of context-dependency in plant response to inoculation with mycorrhizal fungi. Ecol Lett. 2010; 13: 394–407. 10.1111/j.1461-0248.2009.01430.x 20100237

[6] Koziol L, Bever JD. The missing link in grassland restoration: Arbuscular mycorrhizal fungi inoculation increases plant diversity and accelerates succession. J Appl Ecol. 2017; 54: 1301–1309.

[7] Koziol L, Crews TE, Bever JD. Benefits of native mycorrhizal amendments to perennial agroecosystems increases with field inoculation density. Agronomy. 2019; 9: 353.

[8] Cheeke TE, Zheng C, Koziol L, Gurholt CR, Bever JD. Sensitivity to AMF species is greater in late-successional than early-successional native or nonnative grassland plants. Ecology. 2010; 12: e02855 10.1002/ecy.2855 PMC6916349 31359432

[9] Abbott LK, Robson AD. Factors influencing the occurrence of vesicular-arbuscular mycorrhizas. Agric Ecosyst Environ. 1991; 35: 121–150.

[10] Mbuthia LW, Acosta-Martínez V, DeBruyn J, Schaeffer S, Tyler D, Odoi E, et al Long term tillage, cover crop, and fertilization effects on microbial community structure, activity: Implications for soil quality. Soil Biol Biochem. 2015; 89: 24–34.

[11] Oehl F, Sieverding E, Ineichen K, Ris E-A, Boller T, Wiemken A. Community structure of arbuscular mycorrhizal fungi at different soil depths in extensively and intensively managed agroecosystems. New Phytol. 2005; 165: 273–283. 10.1111/j.1469-8137.2004.01235.x 15720639

[12] Jansa J, Mozafar A, Anken T, Ruh R, Sanders I, Frossard E. Diversity and structure of AMF communities as affected by tillage in a temperate soil. Mycorrhiza. 2002;12: 225–234. 10.1007/s00572-002-0163-z 12375133

[13] Oehl F, Sieverding E, Ineichen K, Mäder P, Boller T, Wiemken A. Impact of land use intensity on the species diversity of arbuscular mycorrhizal fungi in agroecosystems of central Europe. Appl Environ Microbiol. 2003; 69: 2816–2824. 10.1128/aem.69.5.2816-2824.2003 12732553 PMC154529

[14] Ryan MH, Chilvers GA, Dumaresq DC. Colonisation of wheat by VA-mycorrhizal fungi was found to be higher on a farm managed in an organic manner than on a conventional neighbour. Plant Soil. 1994; 160: 33–40.

[15] Johnson NC. Can fertilization of soil select less mutualistic mycorrhizae? Ecol Appl. 1993; 3: 749–757. 10.2307/1942106 27759303

[16] House GL, Bever JD. Disturbance reduces the differentiation of mycorrhizal fungal communities in grasslands along a precipitation gradient. Ecol Appl. 2018; 28: 736–748. 10.1002/eap.1681 29314434

[17] Bauer JT, Mack KML, Bever JD. Plant-soil feedbacks as drivers of succession: Evidence from remnant and restored tallgrass prairies. Ecosphere. 2015; 6(9): 1–12.

[18] Putten Van der. Plant defense belowground and spatiotemporal processes in natural vegetation. Ecology. 2003; 84: 2269–2280.

[19] Vukicevich E, Lowery T, Bowen P, Úrbez-Torres JR, Hart M. Cover crops to increase soil microbial diversity and mitigate decline in perennial agriculture. A review. Agron Sustain Dev. 2016; 36: 48.

[20] Bever JD, Westover KM, Antonovics J. Incorporating the soil community into plant population dynamics: The utility of the feedback approach. J Ecol. 1997; 561–573.

[21] Peralta AL, Sun Y, McDaniel MD, Lennon JT. Crop rotational diversity increases disease suppressive capacity of soil microbiomes. Ecosphere. 2018; 9: e02235.

[22] Ryan MR, Crews TE, Culman SW, DeHaan LR, Hayes RC, Jungers JM, et al Managing for Multifunctionality in Perennial Grain Crops. BioScience. 2018; 68: 294–304. 10.1093/biosci/biy014 29662249 PMC5894082

[23] Crews TE, Cattani DJ. Strategies, advances, and challenges in breeding perennial grain crops. Sustainability. 2018; 10: 2192.

[24] Tilman D, Isbell F, Cowles JM. Biodiversity and ecosystem functioning. Annu Rev Ecol Evol Syst. 2014; 45: 471–493.

[25] Isbell F, Adler PR, Eisenhauer N, Fornara D, Kimmel K, Kremen C, et al Benefits of increasing plant diversity in sustainable agroecosystems. J Ecol. 2017; 105: 871–879.

[26] Vandermeer JH. The Ecology of Intercropping. Cambridge University Press; 1992.

[27] Tilman D, Lehman CL, Thomson KT. Plant diversity and ecosystem productivity: Theoretical considerations. Proc Natl Acad Sci. 1997; 94: 1857–1861. 10.1073/pnas.94.5.1857 11038606 PMC20007

[28] Zhu J, Werf W van der, Vos J, Anten NPR, Putten PEL van der, Evers JB. High productivity of wheat intercropped with maize is associated with plant architectural responses. Ann Appl Biol. 2016; 168: 357–372.

[29] Fridley JD. Resource availability dominates and alters the relationship between species diversity and ecosystem productivity in experimental plant communities. Oecologia. 2002; 132: 271–277. 10.1007/s00442-002-0965-x 28547362

[30] Li Q-Z, Sun J-H, Wei X-J, Christie P, Zhang F-S, Li L. Overyielding and interspecific interactions mediated by nitrogen fertilization in strip intercropping of maize with faba bean, wheat and barley. Plant Soil. 2011; 339: 147–161.

[31] Nyfeler D, Huguenin‐Elie O, Suter M, Frossard E, Connolly J, Lüscher A. Strong mixture effects among four species in fertilized agricultural grassland led to persistent and consistent transgressive overyielding. J Appl Ecol. 2013; 683–691.

[32] DeHaan LR, Weisberg S, Tilman D, Fornara D. Agricultural and biofuel implications of a species diversity experiment with native perennial grassland plants. Agric Ecosyst Environ. 2010; 137: 33–38.

[33] Finney DM, Kaye JP. Functional diversity in cover crop polycultures increases multifunctionality of an agricultural system. J Appl Ecol. 2017; 54: 509–517.

[34] Connolly J, Cadotte MW, Brophy C, Dooley Á, Finn J, Kirwan L, et al Phylogenetically diverse grasslands are associated with pairwise interspecific processes that increase biomass. Ecology. 2011; 92: 1385–1392. 10.1890/10-2270.1 21870611

[35] Wang GZ, Li HG, Christie P, Zhang FS, Zhang JL, Bever JD. Plant-soil feedback contributes to intercropping overyielding by reducing the negative effect of take-all on wheat and compensating the growth of faba bean. Plant Soil. 2017; 415: 1–12.

[36] Wang G, Schultz P, Tipton A, Zhang J, Zhang F, Bever JD. Soil microbiome mediates positive plant diversity-productivity relationships in late successional grassland species. Ecol Lett. 2019; 22: 1221–1232. 10.1111/ele.13273 31131969

[37] Wang G, Ye C, Zhang J, Koziol L, Bever JD, Li X. Asymmetric facilitation induced by inoculation with arbuscular mycorrhizal fungi leads to overyielding in maize/faba bean intercropping. J Plant Interact. 2019; 14: 10–20.

[38] Duchene O, Vian J-F, Celette F. Intercropping with legume for agroecological cropping systems: Complementarity and facilitation processes and the importance of soil microorganisms. A review. Agric Ecosyst Environ. 2017; 240: 148–161.

[39] Smith SE, Read DJ. Mycorrhizal Symbiosis. Academic Press; 2010.

[40] Phillips RP, Brzostek E, Midgley MG. The mycorrhizal-associated nutrient economy: a new framework for predicting carbon–nutrient couplings in temperate forests. New Phytol. 2013; 41–51. 10.1111/nph.12221 23713553

[41] Bowles TM, Jackson LE, Cavagnaro TR. Mycorrhizal fungi enhance plant nutrient acquisition and modulate nitrogen loss with variable water regimes. Glob Change Biol. 2018; 24: e171–e182. 10.1111/gcb.13884 28862782

[42] Augé RM. Water relations, drought and vesicular-arbuscular mycorrhizal symbiosis. Mycorrhiza. 2001; 11: 3–42.

[43] Augé RM, Toler HD, Saxton AM. Arbuscular mycorrhizal symbiosis alters stomatal conductance of host plants more under drought than under amply watered conditions: A meta-analysis. Mycorrhiza. 2015; 25: 13–24. 10.1007/s00572-014-0585-4 24831020

[44] Quiroga G, Erice G, Ding L, Chaumont F, Aroca R, Ruiz‐Lozano JM. The arbuscular mycorrhizal symbiosis regulates aquaporins activity and improves root cell water permeability in maize plants subjected to water stress. Plant Cell Environ. 2019; 42: 2274–2290. 10.1111/pce.13551 30916398

[45] Wagg C, Jansa J, Stadler M, Schmid B, Heijden MGA van der. Mycorrhizal fungal identity and diversity relaxes plant–plant competition. Ecology. 2011; 92: 1303–1313. 10.1890/10-1915.1 21797158

[46] Bever JD, Mangan SA, Alexander HM. Maintenance of plant species diversity by pathogens. Annu Rev Ecol Evol Syst. 2015; 46: 305–325.

[47] Schnitzer SA, Klironomos JN, HilleRisLambers J, Kinkel LL, Reich PB, Xiao K, et al Soil microbes drive the classic plant diversity–productivity pattern. Ecology. 2011; 92: 296–303. 10.1890/10-0773.1 21618909

[48] Vukicevich E, Lowery T, Bowen P, Úrbez-Torres JR, Hart M. Cover crops to increase soil microbial diversity and mitigate decline in perennial agriculture. A review. Agron Sustain Dev. 2016; 36: 48.

[49] Maron JL, Marler M, Klironomos JN, Cleveland CC. Soil fungal pathogens and the relationship between plant diversity and productivity. Ecol Lett. 2011; 14: 36–41. 10.1111/j.1461-0248.2010.01547.x 21073641

[50] Putten WH van der, Bradford MA, Brinkman EP, Voorde TFJ van de, Veen GF. Where, when and how plant–soil feedback matters in a changing world. Funct Ecol. 2016; 30: 1109–1121.

[51] Pandey P, Irulappan V, Bagavathiannan MV, Senthil-Kumar M. Impact of combined abiotic and biotic stresses on plant growth and avenues for crop improvement by exploiting physio-morphological traits. Front Plant Sci. 2017; 8. 10.3389/fpls.2017.00537 PMC5394115 28458674

[52] DeHaan L, Christians M, Crain J, Poland J. Development and evolution of an intermediate wheatgrass domestication program. Sustainability. 2018; 10: 1499.

[53] Van Tassel DL, Albrecht KA, Bever JD, Boe AA, Brandvain Y, Crews TE, et al Accelerating *Silphium* domestication: An opportunity to develop new crop ideotypes and breeding strategies informed by multiple disciplines. Crop Sci. 2017; 57: 1274–1284.

[54] Brinkman Pernilla E, Van der Putten WH, Bakker E, Verhoeven KJ. Plant–soil feedback: Experimental approaches, statistical analyses and ecological interpretations. J Ecol. 2010; 98: 1063–1073.

[55] Soil Survey Staff, Natural Resources Conservation Service, United States Department of Agriculture. Web Soil Survey. Available online at http://websoilsurvey.nrcs.usda.gov/. Accessed April 20, 2020].

[56] Koziol L, Bever JD. The missing link in grassland restoration: Arbuscular mycorrhizal fungi inoculation increases plant diversity and accelerates succession. J Appl Ecol. 2017; 54: 1301–1309.

[57] McGonigle TP, Miller MH, Evans DG, Fairchild GL, Swan JA. A new method which gives an objective measure of colonization of roots by vesicular—arbuscular mycorrhizal fungi. New Phytol. 1990; 115: 495–501. 10.1111/j.1469-8137.1990.tb00476.x 33874272

[58] Middleton EL, Richardson S, Koziol L, Palmer CE, Yermakov Z, Henning JA, et al Locally adapted arbuscular mycorrhizal fungi improve vigor and resistance to herbivory of native prairie plant species. Ecosphere. 2015; 6: 1–16.

[59] Bever JD, Platt TG, Morton ER. Microbial population and community dynamics on plant roots and their feedbacks on plant communities. Annu Rev Microbiol. 2012; 66: 265–283. 10.1146/annurev-micro-092611-150107 22726216 PMC3525954

[60] Koziol L, Bever JD. AMF, phylogeny, and succession: Specificity of response to mycorrhizal fungi increases for late-successional plants. Ecosphere. 2016; 7: e01555.

[61] Seifert EK, Bever JD, Maron JL. Evidence for the evolution of reduced mycorrhizal dependence during plant invasion. Ecology. 2009; 90: 1055–1062. 10.1890/08-0419.1 19449699

[62] Kulmatiski A. Community-level plant–soil feedbacks explain landscape distribution of native and non-native plants. Ecol Evol. 2018; 8: 2041–2049. 10.1002/ece3.3649 29468023 PMC5817120

[63] Crawford KM, Bauer JT, Comita LS, Eppinga MB, Johnson DJ, Mangan SA, et al When and where plant-soil feedback may promote plant coexistence: A meta-analysis. Ecol Lett. 2019; 22: 1274–1284. 10.1111/ele.13278 31149765

[64] Turner MK, Ravetta D, Van Tassel D. Effect of *Puccinia silphii* on yield components and leaf physiology in *Silphium integrifolium*: Lessons for the domestication of a perennial oilseed crop. Sustainability. 2018; 10: 696.

[65] Samac DA, Rhodes LH, Lamp WO, editors. Compendium of alfalfa diseases and pests, Third Edition The American Phytopathological Society; 2016.

[66] Diez JM, Dickie I, Edwards G, Hulme PE, Sullivan JJ, Duncan RP. Negative soil feedbacks accumulate over time for non-native plant species. Ecol Lett. 2010; 13: 803–809. 10.1111/j.1461-0248.2010.01474.x 20482584

[67] Bever JD, Dickie IA, Facelli E, Facelli JM, Klironomos J, Moora M, et al Rooting theories of plant community ecology in microbial interactions. Trends Ecol Evol. 2010; 25: 468–478. 10.1016/j.tree.2010.05.004 20557974 PMC2921684

[68] Fargione J, Tilman D, Dybzinski R, Lambers JHR, Clark C, Harpole WS, et al From selection to complementarity: Shifts in the causes of biodiversity–productivity relationships in a long-term biodiversity experiment. Proc R Soc B Biol Sci. 2007; 274: 871–876. 10.1098/rspb.2006.0351 PMC2093979 17251113

[69] Tautges NE, Jungers JM, DeHaan LR, Wyse DL, Sheaffer CC. Maintaining grain yields of the perennial cereal intermediate wheatgrass in monoculture v. bi-culture with alfalfa in the Upper Midwestern USA. J Agric Sci. 2018; 156: 758–773.

