# Supplementary material for: Abiotic and biotic context dependency of perennial crop yield
Source: PLoS One. 2020 Jun 26;15(6):e0234546. doi: 10.1371/journal.pone.0234546 (PMC7319328; doi:10.1371/journal.pone.0234546)
Supplement: S1 Table — (DOCX) [file pone.0234546.s001.docx]

**Table S1.** Degrees of freedom (numerator (NUM) and denominator (Den)), F value, and P value from analysis of the effect of water, crop species (identity), and soil inoculum on the presence of AM fungal hyphae and arbuscules.

|  |  |  | Hyphae | | |  | Arbuscules | | |
| --- | --- | --- | --- | --- | --- | --- | --- | --- | --- |
| Effect | Num |  | Den | F | P |  | Den | F | P |
| Block | 3 |  | 15.1 | 2.71 | 0.0820 |  | 3.0 | 1.55 | 0.3637 |
| Water (W) | 1 |  | 15.1 | 13.52 | 0.0022 |  | 3.0 | 4.58 | 0.1218 |
| Crop Identity (CI) | 2 |  | 14.6 | 6.70 | 0.0086 |  | 46.3 | 11.17 | 0.0001 |
| Inoculum (I) | 2 |  | 20.7 | 35.74 | <0.0001 |  | 18.5 | 9.99 | 0.0004 |
| W x CI | 3 |  | 14.6 | 3.98 | 0.0416 |  | 46.3 | 2.36 | 0.1060 |
| W x I | 3 |  | 20.7 | 2.55 | 0.0835 |  | 18.5 | 1.21 | 0.3331 |
| I x CI | 6 |  | 34.6 | 1.19 | 0.3335 |  | 46.3 | 1.94 | 0.0934 |
| W X I X CI | 6 |  | 34.6 | 1.19 | 0.3337 |  | 46.3 | 0.71 | 0.6449 |
