# Supplementary material for: Abiotic and biotic context dependency of perennial crop yield
Source: PLoS One. 2020 Jun 26;15(6):e0234546. doi: 10.1371/journal.pone.0234546 (PMC7319328; doi:10.1371/journal.pone.0234546)
Supplement: S2 Table — (DOCX) [file pone.0234546.s002.docx]

**Table S2.** Degrees of freedom (numerator (NUM) and denominator (Den)), F value, and p value from analysis of the effect of water, crop community, crop species nested within crop community, and soil inoculum on total average yield per of each crop (Kernza, alfalfa, and *Silphium*) in the first, second, and total harvest.

|  | |  |  | Harvest 1 | | |  | Harvest 2 | | |  | Total Harvest | | |
| --- | --- | --- | --- | --- | --- | --- | --- | --- | --- | --- | --- | --- | --- | --- |
| Effect | Num | |  | Den | F | P |  | Den | F | P |  | Den | F | P |
| Block | | 6 |  | 6.01 | 3.44 | 0.0789 |  | 6.00 | 0.23 | 0.9514 |  | 6.02 | 1.26 | 0.3915 |
| Water (W) | | 1 |  | 6.09 | 165.65 | <0.0001 |  | 6.03 | 58.25 | 0.0003 |  | 6.06 | 115.87 | <0.0001 |
| Crop Community (C) | | 5 |  | 418.00 | 18.19 | <0.0001 |  | 58.30 | 17.33 | <0.0001 |  | 385.00 | 26.51 | <0.0001 |
| Inoculum (I) | | 3 |  | 418.00 | 223.03 | <0.0001 |  | 38.10 | 36.06 | <0.0001 |  | 38.60 | 253.14 | <0.0001 |
| W x C | | 5 |  | 418.00 | 1.22 | 0.2970 |  | 58.30 | 7.32 | <0.0001 |  | 385.00 | 3.86 | 0.0020 |
| W x I | | 3 |  | 418.00 | 39.04 | <0.0001 |  | 38.10 | 20.16 | <0.0001 |  | 38.60 | 53.78 | <0.0001 |
| C x I | | 15 |  | 418.00 | 30.81 | <0.0001 |  | 331.00 | 7.74 | <0.0001 |  | 384.00 | 33.26 | <0.0001 |
| C x crop species (S) | | 3 |  | 418.00 | 117.21 | <0.0001 |  | 328.00 | 93.23 | <0.0001 |  | 383.00 | 149.74 | <0.0001 |
| I x C x S | | 9 |  | 418.00 | 59.38 | <0.0001 |  | 328.00 | 25.19 | <0.0001 |  | 383.00 | 71.66 | <0.0001 |
| W x I x C | | 15 |  | 418.00 | 3.86 | <0.0001 |  | 331.00 | 3.16 | <0.0001 |  | 384.00 | 4.92 | <0.0001 |
| W x C x S | | 3 |  | 418.00 | 2.64 | 0.0492 |  | 328.00 | 40.21 | <0.0001 |  | 383.00 | 19.56 | <0.0001 |
| W x I x C x S | | 9 |  | 418.00 | 8.73 | <0.0001 |  | 328.00 | 11.80 | <0.0001 |  | 383.00 | 13.28 | <0.0001 |
