# Supplementary material for: Abiotic and biotic context dependency of perennial crop yield
Source: PLoS One. 2020 Jun 26;15(6):e0234546. doi: 10.1371/journal.pone.0234546 (PMC7319328; doi:10.1371/journal.pone.0234546)
Supplement: S3 Table — Degrees of freedom (numerator (Num), denominator (Den)), F value, and P value, for planned contrasts to test for overyielding in mixtures (mix) relative to component monocultures (mono) of Kernza (K), alfalfa (A), and Silphium (S) with different soil inoculum present and changing water availability for the first, second, and total harvest. Live represents the presence of native AM fungi, whole soil, or both. (DOCX) [file pone.0234546.s003.docx]

**Table S3.** Degrees of freedom (numerator (Num), denominator (Den)), F value, and P value, for planned contrasts to test for overyielding in mixtures (mix) relative to component monocultures (mono) of Kernza (K), alfalfa (A), and *Silphium* (S) with different soil inoculum present and changing water availability for the first, second, and total harvest. Live represents the presence of native AM fungi, whole soil, or both.

|  |  |  |  | Harvest1 | | |  | Harvest2 | | |  | Total Harvest | | |
| --- | --- | --- | --- | --- | --- | --- | --- | --- | --- | --- | --- | --- | --- | --- |
| Contrast set | Contrast | Num |  | Den | F | P |  | Den | F | P |  | Den | F | P |
| 1 | mix vs mono overall | 1 |  | 418 | 10.38 | 0.0014 |  | 74 | 7.15 | 0.0092 |  | 384 | 12.85 | 0.0004 |
| 1 | mix vs mono KA | 1 |  | 418 | 16.46 | <0.0001 |  | 59.4 | 9.46 | 0.0032 |  | 382 | 17.45 | <0.0001 |
| 1 | mix vs mono KS | 1 |  | 418 | 12.54 | 0.0004 |  | 60.8 | 3.64 | 0.0613 |  | 384 | 11.85 | 0.0006 |
| 1 | mix vs mono AS | 1 |  | 418 | 0.59 | 0.4441 |  | 60.6 | 0.44 | 0.5093 |  | 388 | 0.00 | 0.9829 |
| 2 | mix vs mono overall x whole soil | 1 |  | 418 | 2.62 | 0.1063 |  | 331 | 0.00 | 0.9750 |  | 384 | 0.96 | 0.3272 |
| 2 | mix vs mono overall x AM fungi | 1 |  | 418 | 3.87 | 0.0497 |  | 331 | 0.68 | 0.4096 |  | 384 | 2.51 | 0.1142 |
| 2 | mix vs mono x live vs sterile | 1 |  | 418 | 12.00 | 0.0006 |  | 331 | 0.54 | 0.4638 |  | 383 | 6.46 | 0.0114 |
| 2 | mix vs mono KA x whole soil | 1 |  | 418 | 0.71 | 0.4012 |  | 327 | 0.00 | 0.9465 |  | 382 | 0.07 | 0.7850 |
| 2 | mix vs mono KA x AM fungi | 1 |  | 418 | 4.26 | 0.0397 |  | 327 | 0.00 | 0.9885 |  | 382 | 1.56 | 0.2126 |
| 2 | mix vs mono KA x live vs sterile | 1 |  | 418 | 10.73 | 0.0011 |  | 327 | 0.08 | 0.7714 |  | 382 | 4.25 | 0.0398 |
| 2 | mix vs mono KS overall | 3 |  | 418 | 3.97 | 0.0082 |  | 331 | 2.30 | 0.0773 |  | 384 | 4.51 | 0.0040 |
| 2 | mix vs mono KS x whole soil | 1 |  | 418 | 3.37 | 0.0671 |  | 331 | 0.07 | 0.7915 |  | 384 | 2.16 | 0.1429 |
| 2 | mix vs mono KS x AM fungi | 1 |  | 418 | 5.34 | 0.0213 |  | 332 | 5.30 | 0.0219 |  | 384 | 7.43 | 0.0067 |
| 2 | mix vs mono KS x live vs sterile | 1 |  | 418 | 11.80 | 0.0007 |  | 338 | 4.76 | 0.0297 |  | 387 | 12.73 | 0.0004 |
| 2 | mix vs mono AS overall | 3 |  | 418 | 0.26 | 0.854 |  | 336 | 0.44 | 0.726 |  | 388 | 0.19 | 0.9056 |
| 2 | mix vs mono AS x whole soil | 1 |  | 418 | 0.56 | 0.456 |  | 337 | 0.02 | 0.8954 |  | 388 | 0.11 | 0.7407 |
| 2 | mix vs mono AS x AM fungi | 1 |  | 418 | 0.04 | 0.8377 |  | 337 | 0.33 | 0.5667 |  | 388 | 0.39 | 0.5343 |
| 2 | mix vs mono AS x live vs sterile | 1 |  | 418 | 0.34 | 0.5623 |  | 333 | 0.96 | 0.3283 |  | 386 | 0.10 | 0.7513 |
| 3 | mix vs mono overall x water | 1 |  | 418 | 0.44 | 0.5092 |  | 74 | 1.75 | 0.1903 |  | 384 | 1.30 | 0.2557 |
| 3 | mix vs mono KA x water | 1 |  | 418 | 1.47 | 0.2268 |  | 59.4 | 5.02 | 0.0289 |  | 382 | 3.61 | 0.0583 |
| 3 | mix vs mono KS x water | 1 |  | 418 | 0.26 | 0.6132 |  | 60.8 | 0.00 | 0.948 |  | 384 | 0.09 | 0.7589 |
| 3 | mix vs mono AS x water | 1 |  | 418 | 0.10 | 0.7545 |  | 60.6 | 0.39 | 0.537 |  | 388 | 0.04 | 0.8334 |
| 4 | mix vs mono overall x AM fungi x water | 1 |  | 418 | 0.24 | 0.6216 |  | 331 | 0.01 | 0.9275 |  | 384 | 0.07 | 0.7926 |
| 4 | mix vs mono KA x whole soil x water | 1 |  | 418 | 0.00 | 0.985 |  | 327 | 0.26 | 0.6113 |  | 382 | 0.04 | 0.848 |
| 4 | mix vs mono KA x AM fungi x water | 1 |  | 418 | 0.03 | 0.8722 |  | 327 | 0.28 | 0.5989 |  | 382 | 0.11 | 0.7394 |
| 4 | mix vs mono KS x whole soil x water | 1 |  | 418 | 0.20 | 0.6542 |  | 331 | 0.50 | 0.4788 |  | 384 | 0.00 | 0.9534 |
| 4 | mix vs mono KS x AM fungi x water | 1 |  | 418 | 0.47 | 0.4926 |  | 332 | 1.59 | 0.2083 |  | 384 | 1.12 | 0.2903 |
| 4 | mix vs mono AS x whole soil x water | 1 |  | 418 | 0.01 | 0.9347 |  | 337 | 0.03 | 0.8673 |  | 388 | 0.00 | 0.9589 |
| 4 | mix vs mono AS x AM fungi x water | 1 |  | 418 | 0.04 | 0.8448 |  | 337 | 0.3 | 0.5858 |  | 388 | 0.03 | 0.8628 |
