# Supplementary figures and images for: Abiotic and biotic context dependency of perennial crop yield
Source: PLoS One. 2020 Jun 26;15(6):e0234546. doi: 10.1371/journal.pone.0234546 (PMC7319328; doi:10.1371/journal.pone.0234546)

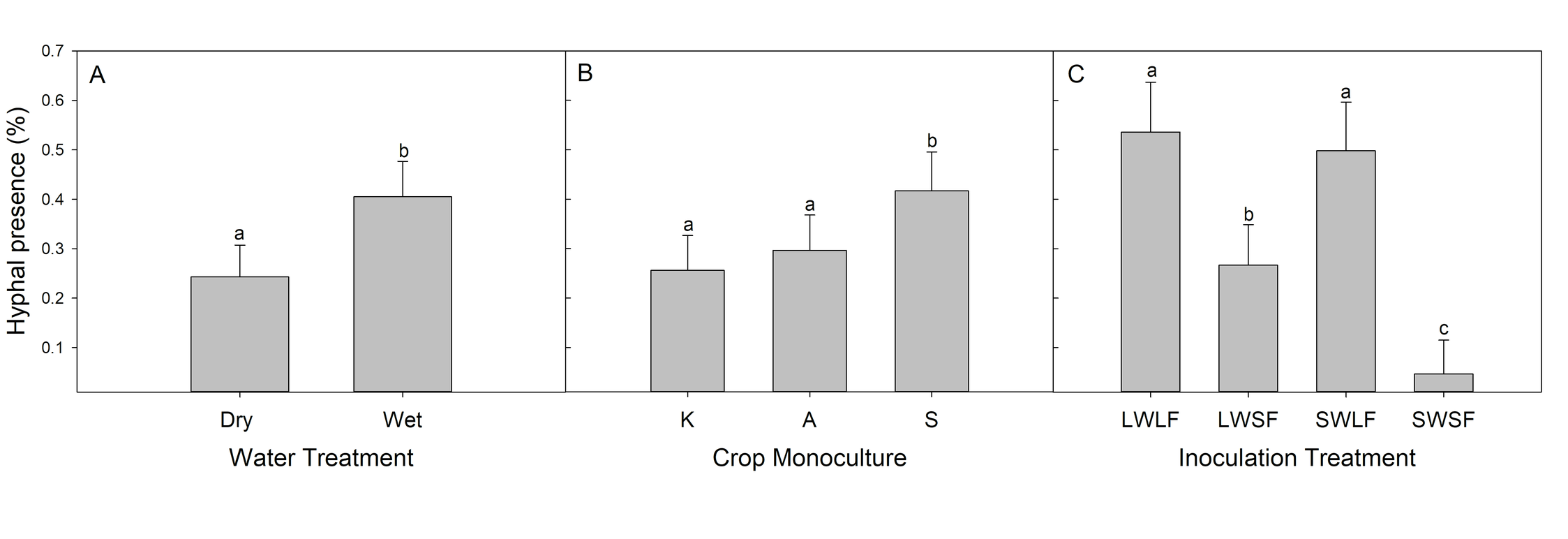

Supplement: S1 Fig — Hyphal colonization (back transformed LS mean ± 95% confidence limits) in monoculture pots with differing watering treatments (A), crop identities (B), and soil inoculation treatments (C). Bars with different letters within each graph are significantly different (Tukey’s HSD multiple comparison). (TIF) [file pone.0234546.s004.tif]

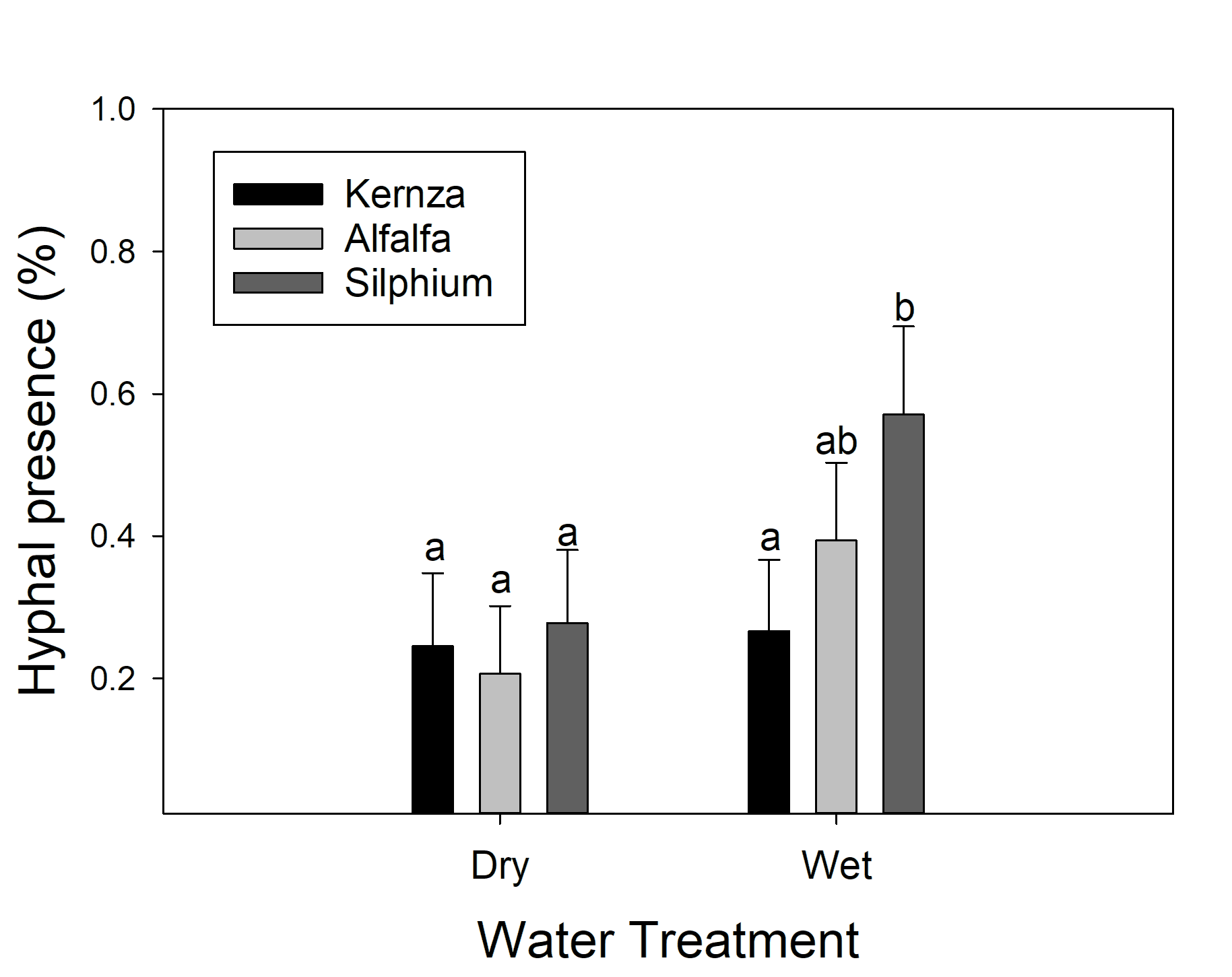

Supplement: S2 Fig — Bars with different letters within each graph are significantly different (Tukey’s HSD multiple comparison). (TIF) [file pone.0234546.s005.tif]

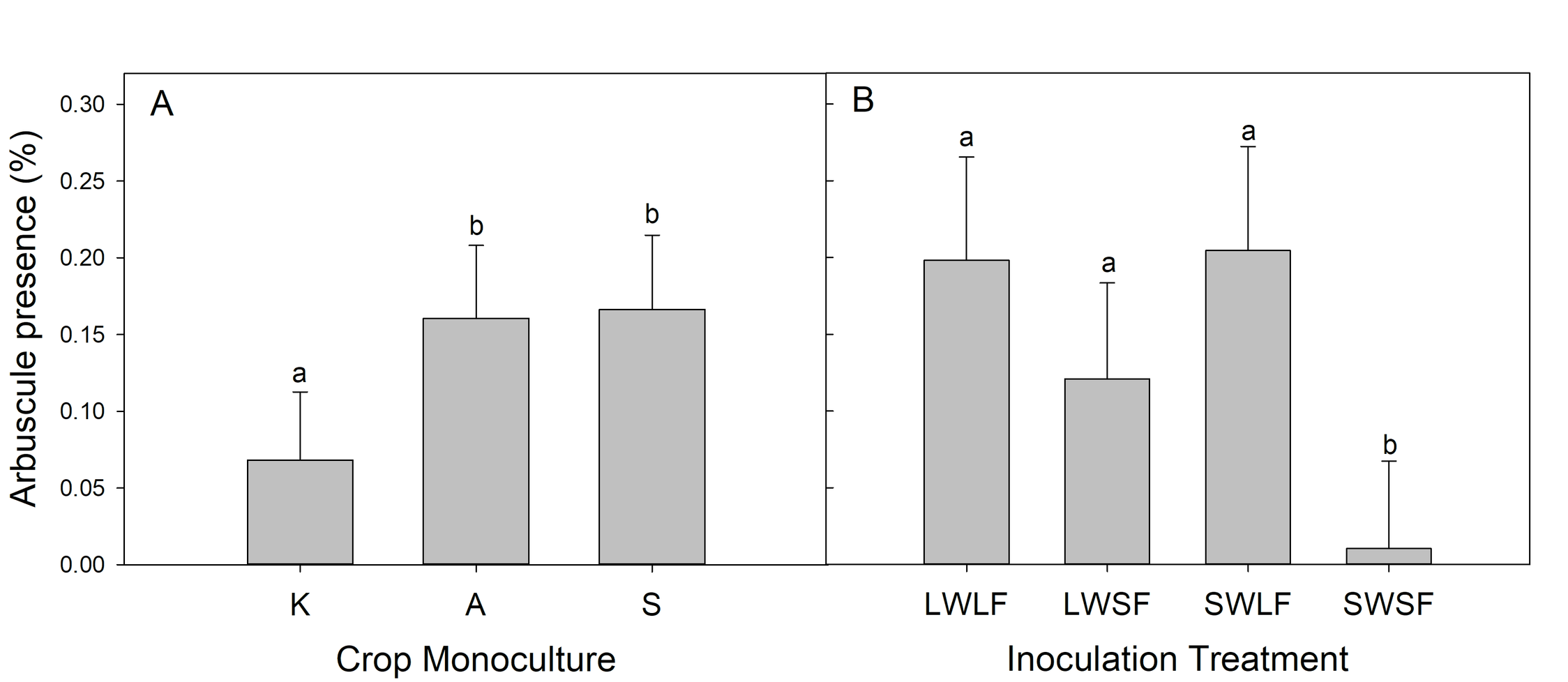

Supplement: S3 Fig — Arbuscule presence (back transformed LS mean ± 95% confidence limits) in monoculture pots with differing crop identities (A) and soil inoculation treatments (B). Bars with different letters within each graph are significantly different (Tukey’s HSD multiple comparison). (TIF) [file pone.0234546.s006.tif]
