# Supplementary material for: Abiotic and biotic context dependency of perennial crop yield
Source: PLoS One. 2020 Jun 26;15(6):e0234546. doi: 10.1371/journal.pone.0234546 (PMC7319328; doi:10.1371/journal.pone.0234546)
Supplement: S1 File — (DOCX) [file pone.0234546.s007.docx]

*AM fungal colonization*

Wet monoculture pots had a greater number of hyphae than dry pots (Table S1; Fig. S1A), and *Silphium* had a greater number of hyphae than Kernza or alfalfa (Fig. S1B). Monocultures inoculated with live prairie AM fungi (LWLF and SWLF) had more hyphae than monocultures with live soil and sterile prairie AM fungi (LWSF), and monocultures with sterile whole soil and sterile prairie AM fungi (SWSF) had the least (Fig. S1C). Water availability moderated the effects of the identity of the crop in monoculture on hyphal presence (water x crop identity interaction; Table S1). In dry pots, hyphal presence was similar across the crop monocultures, but in wet pots *Silphium* monocultures had more hyphae than Kernza monocultures (Fig. S2).

Alfalfa and *Silphium* had a greater number of arbuscules than Kernza (Fig. S3A). Pots with live inoculum (LWLF, LWSF, and SWLF) had more abuscules than sterile pots (SWSF) (Fig. S3B).
